# Bioinformatics identification and integrative analysis of ferroptosis-related key lncRNAs in patients with osteoarthritis

**DOI:** 10.1042/BSR20230255

**Published:** 2023-09-13

**Authors:** Tengyun Yang, Guang Yang, Guoliang Wang, Di Jia, Bohan Xiong, Xiaojun Lu, Yanlin Li

**Affiliations:** Department of Sports Medicine, The First Affiliated Hospital, Kunming Medical University, Kunming 650032, Yunnan, China

**Keywords:** biological markers, ceRNA, ferroptosis, lncRNA, osteoarthritis

## Abstract

Background: Ferroptosis and dysregulation of long non-coding RNA (lncRNA) have been described to be strictly relevant to the pathogenesis of osteoarthritis (OA). However, the connection between ferroptosis and lncRNA in OA is poorly appreciated. Herein, we investigated the functional contribution of lncRNA markers correlated with the progression of human OA by comprehensive bioinformatics analysis of a panoramic network of competing endogenous RNA (ceRNA) based on ferroptosis-related genes (FRGs).

Methods: FRGs-related competing endogenous RNA (ceRNA) networks were generated using differentially expressed genes based on OA-related whole transcriptome data from the Gene Expression Omnibus (GEO) database via starBase, miRTarBase, and miRWalk databases. The pivotal lncRNAs were ascertained by topological features (degree, betweenness, and closeness) and subceRNA networks were re-visualized. The expression difference of pivotal lncRNAs was verified by quantitative real-time polymerase chain reaction (qRT-PCR). The latent molecular mechanisms of the global ceRNA and subceRNA networks were uncovered by the R package clusterProfiler-based enrichment analysis.

Results: A total of 98 dysregulated lncRNA-miRNA-mRNA regulatory relationships were attained in the FRGs-related panoramic ceRNA network of OA, covering 26 mRNAs, 20 miRNAs, and 20 lncRNAs. Three lncRNAs (AC011511.5, AL358072.1, and C9orf139) were ascertained as the central lncRNAs in the panoramic ceRNA network. Functional ensemble analysis illustrated that both the panoramic ceRNA network and the subceRNA network were integrally affiliated with the immune-inflammatory response, oxygen homeostasis, and cell death (apoptosis, autophagy, and ferroptosis).

Conclusion: Comprehensive bioinformatics analysis of the FRGs-related ceRNA network determined three molecular biomarkers of lncRNAs that might be affiliated with OA progression.

## Introduction

Osteoarthritis (OA) is one of the most common chronic diseases, following hypertension, obesity, hypercholesterolemia, and various soft tissue diseases in the 60–69 age group [1]. More than 300 million patients with OA have been diagnosed worldwide [2], and its incidence has risen significantly with the aging of the global population. The condition significantly impacts patients’ quality of life and imposes a substantial economic burden on both patients and society. Consequently, it has become a major global public health concern [3]. OA is a complex disease with various potential causes that may contribute to its development and progression. Some risk factors such as genetic predisposition, aging, obesity, and joint malalignment have been suggested [4,5]. Recent studies have also shown that the infrapatellar fat pad produces adipokines, cytokines, complex fatty acids, and oxidized lipoxins. These may be involved in modulating the pathogenesis of knee osteoarthritis through inflammatory processes. [6–9] Pathologically, OA primarily affects articular cartilage, it is identified by the presence of focal areas of damage in the articular cartilage that are centered on load-bearing areas [10]. This is often accompanied by osteophytosis, or new bone formation at the joint margins, as well as changes in the subchondral bone, varying degrees of mild synovitis, and thickening of the joint capsule [10]. Furthermore, it has been observed that OA also has a non-negligible reflex on the mechanical behavior of articular cartilage due to pathological structural damage. [11,12] This has led to the therapeutic focus on tissue regeneration in OA, together with the need to maintain maximum restoration of mechanical properties. The precise molecular mechanisms of OA are poorly understood, and there is currently no cure to stop or reverse its progression. Therefore, it is crucial to conduct further research to gain a deeper understanding of the pathogenesis of OA. This will help identify more specific targets for effective diagnosis and treatment of OA.

Ferroptosis was first reported by Dixon et al. [13] as a new form of programmed cell death, which was morphologically, biochemically and genetically different from other forms of programmed cell death. Ferroptosis is an iron-dependent non-apoptotic cell death characterized by GPX4 inactivation and lipid-ROS accumulation [13]. Studies have indicated the presence of abnormal iron metabolism in patients with OA in cartilage degeneration [14], and iron overload can accelerate the progression of OA [15]. In addition, altered mitochondrial structure, kinetics and genomic stability can contribute to reduced mitochondrial respiration and overproduction of reactive oxygen species (ROS), ultimately causing oxidative damage to OA chondrocytes [16,17]. Hence, abnormal iron metabolism, lipid peroxidation and mitochondrial dysfunction are all typical features associated with ferroptosis, indicating that ferroptosis may be involved in the regulation of OA. Recent studies have demonstrated that chondrocyte ferroptosis plays a significant role in the development of osteoarthritis [18–20]. It has been reported that the up-regulation of ferroptosis in chondrocytes is significantly induced by IL-1β, while inhibition of ferroptosis promotes chondrocyte survival and cartilage matrix synthesis during the pathological progression of OA [20–22]. Additionally, intra-articular injection of a ferroptosis inhibitor has been shown to ameliorate cartilage damage in a mouse model of OA [23–25]. These suggest that targeting chondrocyte ferroptosis could be a potential therapeutic approach for OA.

Long non-coding RNAs (lncRNAs) defined as more than 200 nucleotides and lacking coding capacity are emerging as key regulators of gene expression [26]. Previous studies have implicated some lncRNAs in the pathogenesis of OA. For instance, lncRNA THUMPD3-AS1 promotes chondrocyte proliferation and inflammatory response in OA [27]. LncRNA h19 attenuates inflammation in OA through the interaction between TP53, IL-38, and IL-36 receptors [28]. In addition, study [29] has shown that the major method lncRNA regulates biological processes is by competitive binding of mRNA to miRNA. LncRNA can bind miRNA competitively, operate as competitive endogenous RNA (ceRNA) to regulate the transcription and expression of downstream genes by minimizing the combination of miRNA and downstream genes thereby controlling biological processes [29]. A recent study suggested that lncRNA Gm37494 could attenuate osteoarthritic chondrocyte injury via microRNA-181a-5p/GABRA1 axis [30]. The lncRNA LEMD1-AS1 attenuated osteoarthritic chondrocyte inflammation by targeting miR-944/PGAP1 [31]. These researches confirmed the importance of lncRNAs as ceRNAs in OA.

However, current studies have focused on autophagy and apoptosis of chondrocytes in OA, and there is no conclusive evidence of a link between OA, lncRNAs, and ferroptosis. LncRNA-mediated association with ferroptosis-related ceRNA network studies in OA remains lacking. In our study, we hypothesized that ferroptosis was involved in the onset and development of OA, which was linked through lncRNAs. The aim of the present study was to explore lncRNAs associated with ferroptosis that had diagnostic value for OA and to build a ceRNA network based on these lncRNAs. We believe such a network could provide a basis for further studies on the specific mechanisms and significance of ferroptosis in OA and provide possible directions for identifying new therapeutic targets.

## Materials and methods

### Data source

GSE114007, GSE143514, and GSE175960 datasets were collected from Gene Expression Omnibus (GEO) database (http://www.ncbi.nlm.nih.gov/geo/) based on the source and size of samples and experiment type. The GSE143514 embraced microRNA (miRNA) analysis of three normal and five OA human knee synovial tissues by a next-generation sequencing tool (https://www.ncbi.nlm.nih.gov/geo/query/acc.cgi?acc=GSE143514) [32]. GSE114007 was a dataset of messenger RNA (mRNA) expression profiles of cartilage tissues from 20 OA patients and 18 normal controls (https://www.ncbi.nlm.nih.gov/geo/query/acc.cgi?acc=GSE114007) [33]. GSE175960 dataset, meanwhile, comprised lncRNA expression profiles of three OA patients and three matched healthy controls (cartilage tissue) (https://www.ncbi.nlm.nih.gov/geo/query/acc.cgi?acc=GSE175960). Both of GSE114007 and GSE175960 were obtained by Microarray analysis. Finally, 259 ferroptosis-related genes (FRGs) (Supplementary Table S1) including Driver, Suppressor, and Marker were obtained from the FerrDb database [34] (http://www.zhounan.org/ferrdb).

### Recognition of exceptionally expressed genes

The aberrant expression probing of mRNA and lncRNA was finished by the R package limma [35], while miRNA was executed by the R package edgeR [36]. For the determination of differentially expressed mRNAs (DE-mRNAs), miRNAs (DE-miRNAs), and lncRNAs (DE-lncRNAs), respectively, the criteria were considered according to the following, DE-mRNA: |log_2_fold change (FC)| > 1 and false discovery rate (FDR) < 0.05; DE-miRNA: |log_2_FC| > 0.5 and *P*<0.05; DE-lncRNA: |log_2_FC| > 1 and *P*<0.05. All comparisons were made in OA versus normal. Furthermore, FRGs in DE-mRNAs were recognized by Jvenn online tool (http://jvenn.toulouse.inra.fr/app/example.html) with cross-tabulation analysis, which were designated as differentially expressed ferroptosis-related genes (DE-FRGs).

### Construction of a panoramic network of the FRGs-related ceRNAs

The relationship pairs of DE-miRNAs-DE-lncRNAs/DE-FRGs were integrated from the starBase [37], miRTarBase [38], and miRWalk [39] databases. Specifically, the starBase database was utilized to predict miRNAs with reciprocal relationships with DE-lncRNAs (denoted as starBase-miRNAs) to obtain candidate DE-lncRNA-starBase-miRNA relationship pairs (denoted as candidate lncRNA-miRNA relationship pairs). Meanwhile, miRNA-DE-FRGs relationship pairs were forecasted by miRTarBase and miRWalk databases, respectively, and then, miRTarBase-miRNA-DE-FRGs relationship pairs and miRWalk-miRNA-DE-FRGs relationship pairs were combined to yield candidate miRTarBase& miRWalk-miRNA-DE-FRGs relationship pairs (noted as candidate miRNA-mRNA relationship pairs). Thereafter, the candidate lncRNA-miRNA-mRNA axis was achieved after eliminating the non-common miRNAs and non-DE-miRNAs from the above candidate lncRNA-miRNA and miRNA-mRNA relationship pairs. We extracted the expression trends of all genes in the candidate lncRNA-miRNA-mRNA axis, and according to the ceRNA regulatory mechanism, the expression trends of lncRNAs and mRNAs were opposite to miRNAs, and the final lncRNA-miRNA-mRNA ceRNA network was attained.

### Analysis of the centrality of DE-lncRNAs in ceRNA networks

The centrality analysis of ceRNA networks was performed by three topological features, including degree, betweenness, and closeness. Among these parameters, the degree is defined as the frequency of connection between a node and other nodes in the network [40], and a larger degree indicates the presence of more complex interactions at that node. Betweenness is a metric to assess the influence of a node and mainly refers to the frequency with which a node disseminates information through the network. Nodes with high betweenness characteristics imply an important role in information dissemination [41]. Closeness points to the measure of the shortest path for a node to access all other proteins in the network [40]. Here, the centrality of lncRNAs in the network was our main concern; therefore, the statistics of three topological features of all lncRNAs were extracted. The common elements of the top 5 genes under the three topological features were screened based on cross-tabulation analysis, and they were considered pivotal DE-lncRNAs.

### Protein interactions of mRNAs in the panoramic ceRNA network

All mRNAs in the panoramic ceRNA network were retrieved and the protein interactions of which were forecasted by the STRING [42] online tool (https://cn.string-db.org/).

### Network symbolization

The panoramic ceRNAs in this study were visualized by Cytoscape [43] software, including subceRNA networks based on critical DE-lncRNAs and the protein–protein interaction (PPI) network of all mRNAs in the panoramic ceRNA network.

### Functional elucidation of the target genes

The corresponding mRNAs in the panoramic ceRNA and subceRNA networks were extracted and functional interpretation was implemented aiming to preliminarily probe the latent mechanisms of ceRNA networks in OA. The analysis was undertaken in the R package clusterProfiler [44] based on Gene Ontology (GO) and Kyoto Encyclopedia of Genes and Genomes (KEGG) databases. Furthermore, the mRNAs in the subceRNA network were also subjected to classical pathway enrichment analysis by Ingenuity Pathway Analysis (IPA). The status of the enriched pathway was judged by *Z*-score, and a positive *Z*-score indicated that the pathway was activated, and vice versa, it was inhibited. The enrichment criterion was established as *P*<0.05.

### Patient preparations

To confirm the outcomes of bioinformatics analysis, we collected cartilage tissues for RT-PCR validation. Cartilage tissue samples were collected from waste cartilage of 10 patients who underwent total knee arthroplasty for osteoarthritis at the First Affiliated Hospital of Kunming Medical University. Patients with a history of ligament rupture, total meniscectomy, joint infection, or systemic rheumatism were excluded from the study. There were 4 males and 6 females, 7 right knees and 3 left knees, with a mean age of 65 (57–78) and a mean body mass index (BMI) of 28.5 kg/m^2^ (24–33 kg/m^2^). Healthy cartilage samples were obtained from the non-weight-bearing part of the lateral femoral area, and the damaged cartilage tissue obtained from the defective area consisted of whole cartilage without subchondral bone according to the method in the literature [45]. Cartilage samples obtained from 10 patients were classified as damaged or healthy non-weight-bearing cartilage. Therefore, there were no significant differences in baseline characteristics two groups of samples. The study protocol was approved by the Ethics Committee of the First Affiliated Hospital of Kunming Medical University (approval number: 2018L12, approval date: 2018-07-25), all patients consented and signed an informed consent form.

### RNA isolation and quantitative real-time polymerase chain reaction (qRT-PCR)

A total of 20 cartilage tissues were frozen in liquid nitrogen for 1 hour, then crushed and homogenised in an enzyme-free sterile mortar. Add 1 ml of TRIzol Reagent (Life Technologies-Invitrogen, U.S.A.) per 100 mg of tissue and homogenise until completely lysed. Transfer to a centrifuge tube.and total RNA was isolated following the manufacturer’s instructions. The concentration and purity of the RNA solution were then determined using a NanoDrop 2000FC-3100 Nucleic Acid Protein Quantificator (Thermo Fisher Scientific, U.S.A.). Prior to qRT-PCR, the extracted RNA was reverse transcribed into cDNA using the Surescript First Strand cDNA Synthesis Kit (Servicebio, China). Quantitative real-time PCR (qRT-PCR) was conducted on the BIO-RAD CFX96 Touch TM PCR detection system (Bio-Rad Laboratories, U.S.A.) with SYBR Green qPCR Master Mix (Genecopoeia, China). The reaction parameters included a denaturation program (1 min at 95°C), followed by an amplification and quantification program over 40 cycles (20 s at 95°C, 20s at 55°C, and 30 s at 72°C). All primers were synthesized by Tsingke (Biotechnology, China) and shown in Table 1.

**Table 1:** Primers for qRT-PCR used in the present study

Primer	Sequence
C9orf139 F	CGTGGTGTGTGAGCATCGT
C9orf139 R	CTCTTGGTCTGGAGTGGGG
AL358072.1 F	TAGTGATGCCACAGGCCAAAT
AL358072.1 R	ATAAACCGCTGCCAACCCAC
AC011511.5 F	CCTTTACGCCTGGGACTTCAT
AC011511.5 R	TTCCTAATTCTCGCCTTCTGC
Internal reference-GAPDH F	ACAACTTTGGTATCGTGGAAGG
Internal reference-GAPDH R	GCCATCACGCCACAGTTTC

Each sample was tested in triplicates, and each sample underwent a melting curve analysis to check for the specificity of amplification. The GAPDH gene served as an internal control, and the relative expression of three pivotal DE-lncRNAs was determined using the 2^−ΔΔCt^ method [46]. Statistical differences of three pivotal DE-lncRNAs between normal and OA samples were detected by unpaired *t*-tests, using GraphPad Prism V6 (GraphPad Software, U.S.A.), and the level of statistical significance was tested and represented as **** for *P*<0.0001.

### Fluorescence *in situ* hybridization (FISH)

Localization of lncRNAs in OA cartilage tissue was detected using the FISH Tag RNA Multicolor Kit (Thermo Fisher Scientific, U.S.A.). LncRNAs probes custom designed by GenePharma (China) were used and shown in Table 2. FISH was performed according to the manufacturer's protocol. Samples were washed with PBS and fixed with 1 ml of 4% PFA for 12 h at room temperature. After dehydration in gradient alcohol, the samples were embedded in paraffin wax, cut into 3-μm slices, dewaxed, and digested with Proteinase K (20 μg/ml) for 12 min at 37°, rinsed in PBS and incubated for 1 h at 37°C. The pre-hybridization solution was discarded and a 500 nM hybridization solution containing probe C9orf139/AC011511.5/AL358072.1 was added. The solution was incubated overnight at 40°C in a constant temperature chamber. The next day, the sections were washed three times with 50% formamide/2× SSC (sodium saline citrate) for 5 min, incubated with DAPI (Servicebio, China) for 5 min protected from light, washed three times with 50% formamide/2× SSC (sodium saline citrate) for 5 min and then sealed with anti-fluorescence quenching blocker (Servicebio, China), and the images were acquired using a Nikon ortho-fluorescence microscope (Nicon Eclipse CI and DS-U3, Japan).

**Table 2:** Probe for FISH used in the present study

Probe	Sequence
C9orf139	5′-TTCTCTTTGACTCATGCCCTTTTCTTT-3′
	5′-GCTTGTGTCCTGATCATCACGATG-3′
	5′-TGGCGGCAGTAAGAGGCAGATTT-3′
	5′-GCCTTCAACTTTGTCCAAGCTCCT-3′
	5′-GTGTGTCCCATCTTCTTCTTGTTCTG-3′
AL358072.1	5′-GTAACCAAATACCACCTGTACCCCA-3′
	5′-CTATCTGGAATAGGGACAGGGGATC-3′
AC011511.5	5′-TTCACTGGGAGCTTGCACTA-3′
	5′-CCTCCCCACCCACATACATT-3′

### Statistical analysis

All open databases and R package were utilized to analyze and visualize in the present study. The boxplot was plotted via ggplot2 package [47]. The heat map was painted using pheatmap package [48]. Comparisons between the two groups were implemented using the Student’s *t*-test. If not specified above, a *P*-value less than 0.05 was considered statistically significant.

## Results

### Authentication of genes aberrantly expressed in OA

**Figure 1:**
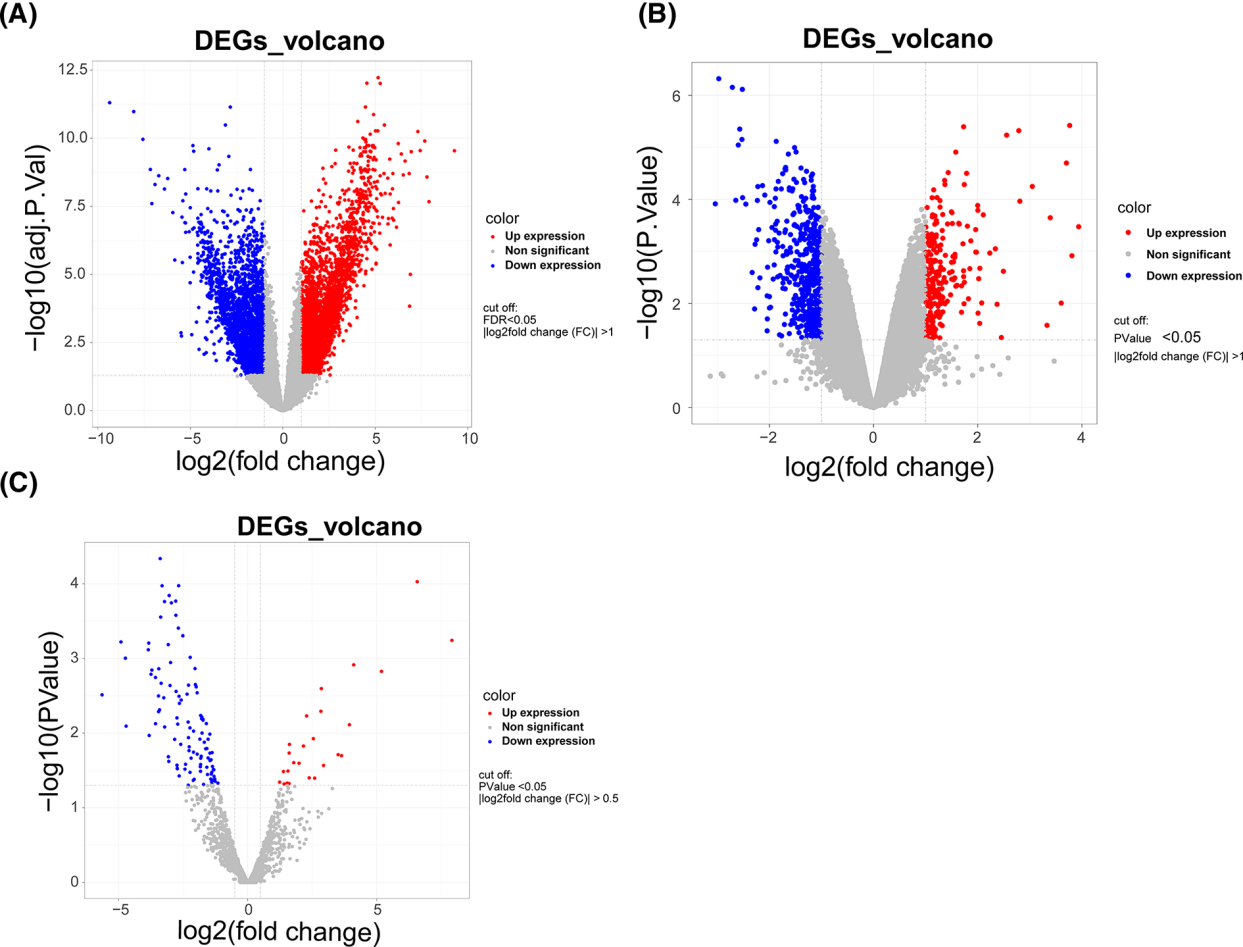
Differentially expressed genes expression volcano plot between osteoarthritis (OA) and normal controls (**A**) Differentially expressed mRNAs were selected by volcano plot filtering. (**B**) Differentially expressed lncRNAs were selected by volcano plot filtering. (**C**) Differentially expressed miRNAs were selected by volcano plot filtering.

The flowchart of the study was displayed in Supplementary Figure S1. An OA versus normal-based variation analysis of gene expression was implemented in the GSE114007-mRNA and GSE143514-miRNA expression profiles with the R package limma. Within the GSE114007 dataset, an aggregate of 5830 DE-mRNAs was recognized (Supplementary Table S2). There were 3291 mRNAs expressed up-regulated (log_2_ FC > 1) and 2539 down-regulated (log_2_ FC < −1) in the OA group (*n*=20) compared with the normal group (*n*=18) (all FDR < 0.05; Figure 1A). In the GSE143514 dataset, at *P*<0.05, a total of 736 DE-lncRNAs satisfied |log_2_ FC| > 1 (Supplementary Table S3). Of these, 265 and 471 were up- and down-regulated, respectively, in the OA population (*n*=5) relative to normal cartilage tissue (*n*=3) (Figure 1B). The determination of 137 DE-miRNAs (|log_2_FC| > 0.5 and *P*<0.05; Supplementary Table S4) was accomplished via R package edgeR based on the GSE175960 dataset. The prevalence of up- and down-regulated genes was 18.25% and 81.75%, respectively (Figure 1C).

### Establishment and visualization of DE-FRGs-based ceRNA network

**Figure 2:**
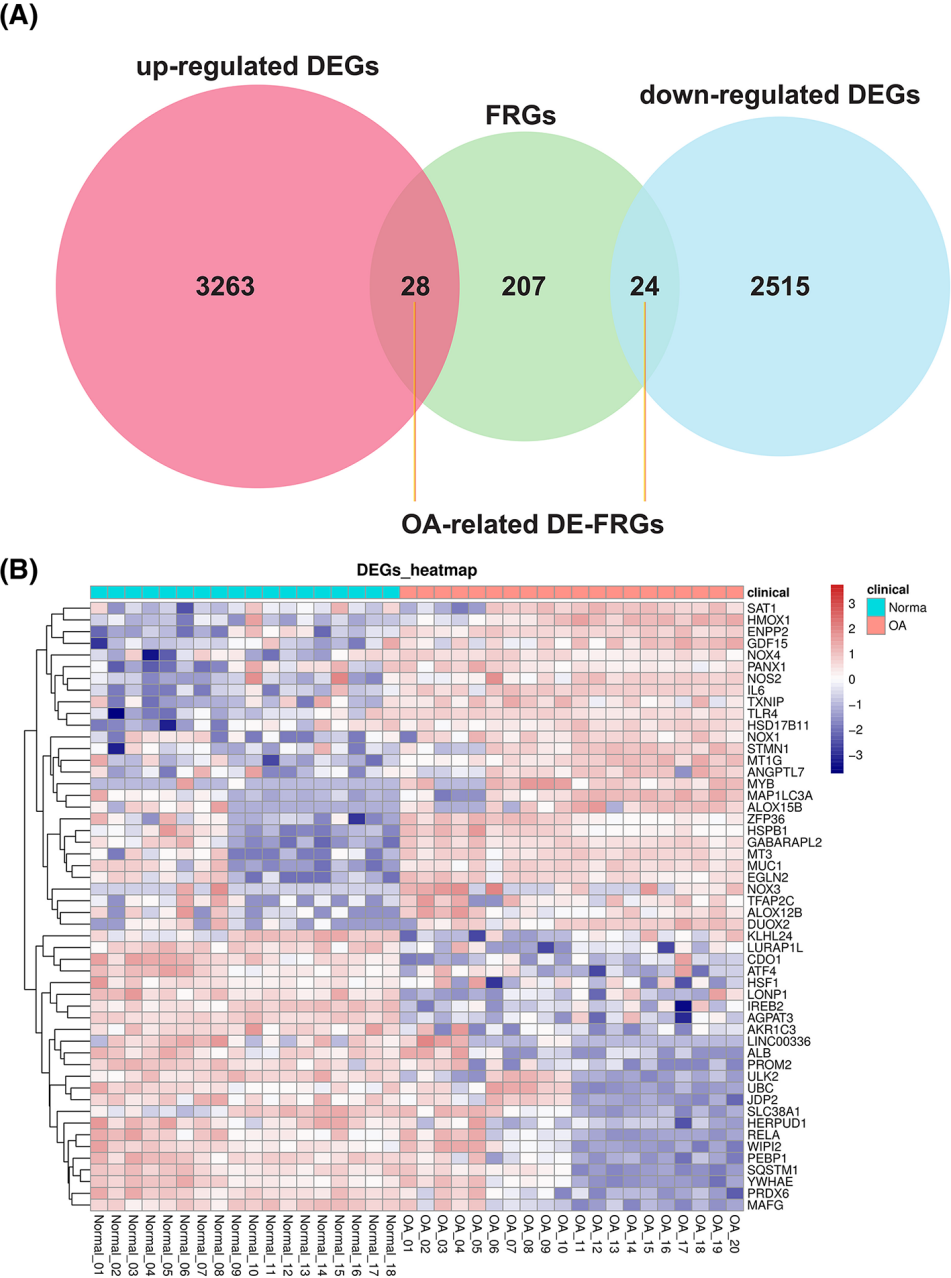
Identification of FRGs (**A**) Venn diagram showing the numbers of overlapped genes between OA-related genes and ferroptosis-related genes (up-regulated genes are marked in red; down-regulated genes are marked in blue). (**B**) Differentially expressed gene expression heatmap of cartilage tissue (all up-regulated and down-regulated genes).

We collimated the expression alterations of 259 FRGs acquired from FerrDb between OA and normal samples by cross-tabulation analysis. The results were presented in Figure 2A, and a total of 52 OA-related DE-FRGs were characterized in the GSE114007 dataset (Supplementary Table S5), of which 28 were expressed up-regulated and 24 down-regulated in OA samples (OA vs. normal; Figure 2B).

**Figure 3:**
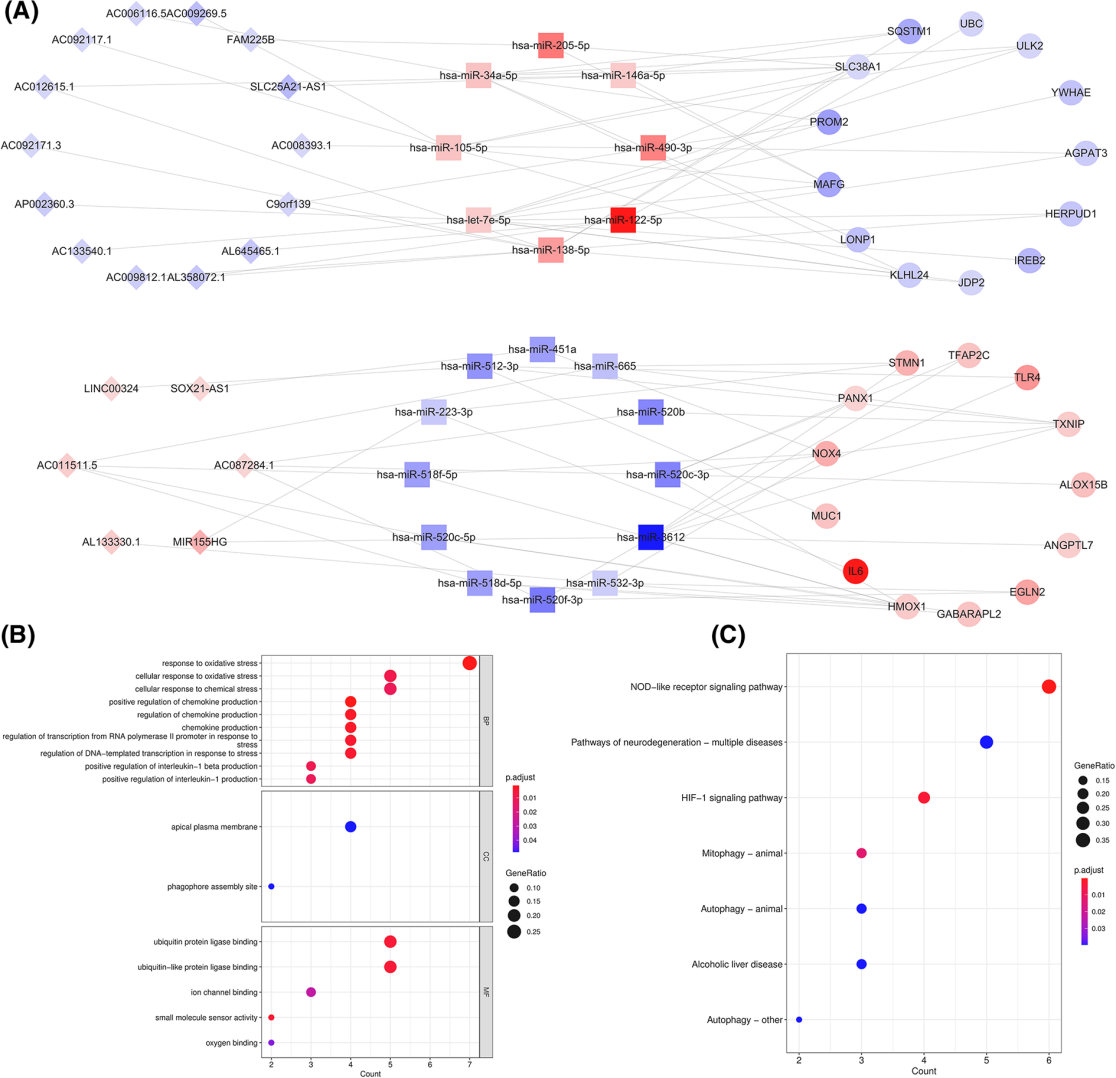
Molecular mechanism of DE-FRGs-based ceRNA network (**A**) CeRNA regulatory network was constructed. The diamond represents lnRNA, the square represents miRNA, and the circle represents mRNA. Blue represents down-regulated genes and red represents up-regulated genes, darker color indicates greater degree the changes have happened of gene expression. (**B**) GO analyses results of DE-FRGs. (**C**) KEGG Pathway analyses results of DE-FRGs (top10 according to adjusted *P-*value).

Subsequently, we predicted the interactions of 91 DE-lncRNAs with 528 starBase-miRNAs from the starBase database. Meanwhile, 14,322 miRNA-mRNA relationship pairs of 51 DE-FRGs and 2,498 miRTarBase and miRWalk-miRNAs were integrated based on miRTarBase and miRWalk databases. Then, we attained 712 lncRNA-miRNA-mRNA axes by merging the above two forecasted regulatory relationships (lncRNA-miRNA and miRNA-mRNA) and discarding non-DE-miRNAs. Eventually, a global view of the OA DE-lncRNA-DE-miRNA-DE-FRGs ceRNA network was structured based on the theory of ceRNA regulatory mechanisms, which was visualized in Figure 3A. The network comprised 98 ceRNA regulatory mechanisms consisting of 20 DE-lncRNAs, 20 DE-miRNAs, and 26 DE-FRGs (Supplementary Table S6), in which 14 DE-lncRNAs were down-regulated in OA samples and 6 of that were up-regulated. Moreover, we analyzed the degree characteristics of the nodes in this network and noticed that most nodes exhibited relatively low degrees and only a few nodes had the higher degrees (Supplementary Figure S2A). Meanwhile, the degree distribution of the nodes appeared a power-law distribution f(*x*) = 24.024*x*∧ (−0.976) with  *R*^2^ of 0.887 (Supplementary Figure S2B), signifying that the FRGs-related ceRNA network adhered to a scale-free distribution [49].

### Molecular mechanism of DE-FRGs-based ceRNA network

To initially probe the possible molecular mechanisms of the FRGs-related ceRNA network in OA progression, we extracted 26 DE-FRGs in this network for functional elucidation reliant on the R package clusterProfiler. Within the GO system, these genes were enclosed with a total of 44 terms, including 37 biological processes (BP), 2 cellular components (CC), and 5 molecular functions (MF) (Supplementary Table S7). Figure 3B captured the top 10 BP entries and all CC and MF entries. Hereby, we were intrigued chiefly by the BP category and found that these genes might be tightly integrated with the immune-inflammatory response (chemokine/interleukin/cytokine production) and intra-articular oxygen homeostasis (response to oxidative stress, hypoxia, and oxygen levels) in the OA cartilage environment. Coherently, the enrichment of the KEGG pathway, NOD-like receptor and HIF-1 signaling pathway further buttressed the hypothesis that the FRGs-related ceRNA network was probably engaged in the immune-inflammatory response and oxygen homeostasis in OA progression. Besides, the autophagy pathway also appeared notably enriched (Figure 3C and Supplementary Table S8).

Furthermore, we structured the PPI network of 26 DE-FRGs in the ceRNA network by STRING tool. After rejecting the discrete proteins, a PPI network containing 16 nodes and 26 edges was visualized with the Cytoscape software (Supplementary Figure S3).

### SubceRNA networks based on the pivotal DE-lncRNAs

**Figure 4:**
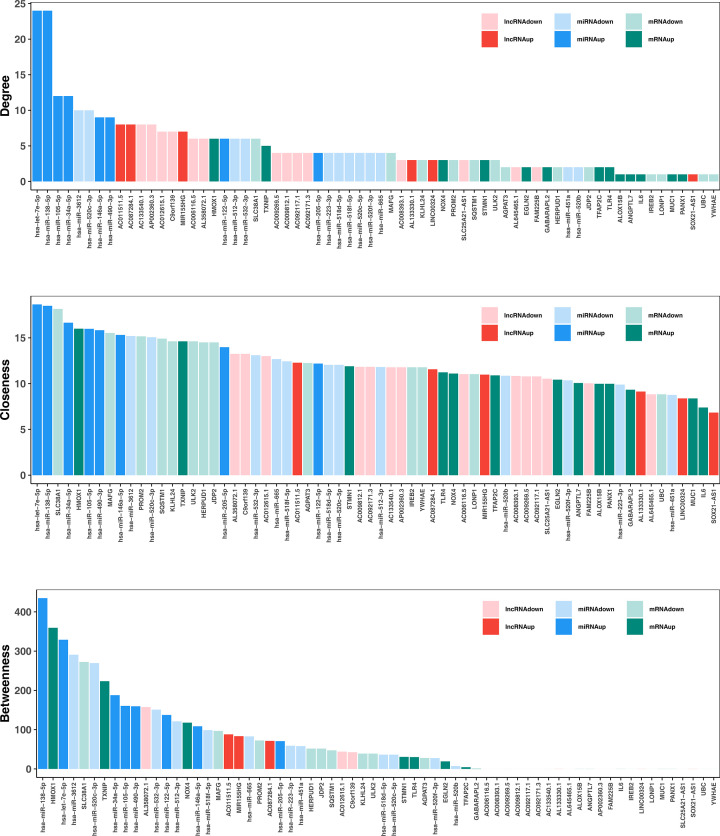
Three topological traits of all nodes in the ceRNA network

This study utilized the topological traits (degree, betweenness, and closeness) of the ceRNA network to pinpoint the important DE-lncRNAs in the network. Three topological traits of all nodes in the ceRNA network were displayed in Figure 4. We detached the three topological traits of nodes with DE-lncRNAs attributes and grabbed the top 5 DE-lncRNAs under each topological trait displayed in Table 3. AC011511.5, AL358072.1, and C9orf139 were the common DE-lncRNAs based on the top 5 elements of the three topological traits, which were designated as pivotal DE-lncRNAs, and their annotation information would be retrieved in Supplementary Table S9.

**Table 3:** The results of the pivotal lncRNAs screening based on topological traits of nodes

Node	Degree	Node	Betweenness	Node	Closeness
AC011511.5	4	C9orf139	17.45	AL358072.1	13.25
AC087284.1	3	MIR155HG	41.1	C9orf139	13.25
AC012615.1	2	AC087284.1	41.41	AC012615.1	13
AL358072.1	2	AC011511.5	56.67	AC011511.5	12.28
C9orf139	2	AL358072.1	115.32	AC009812.1	11.83

**Figure 5:**
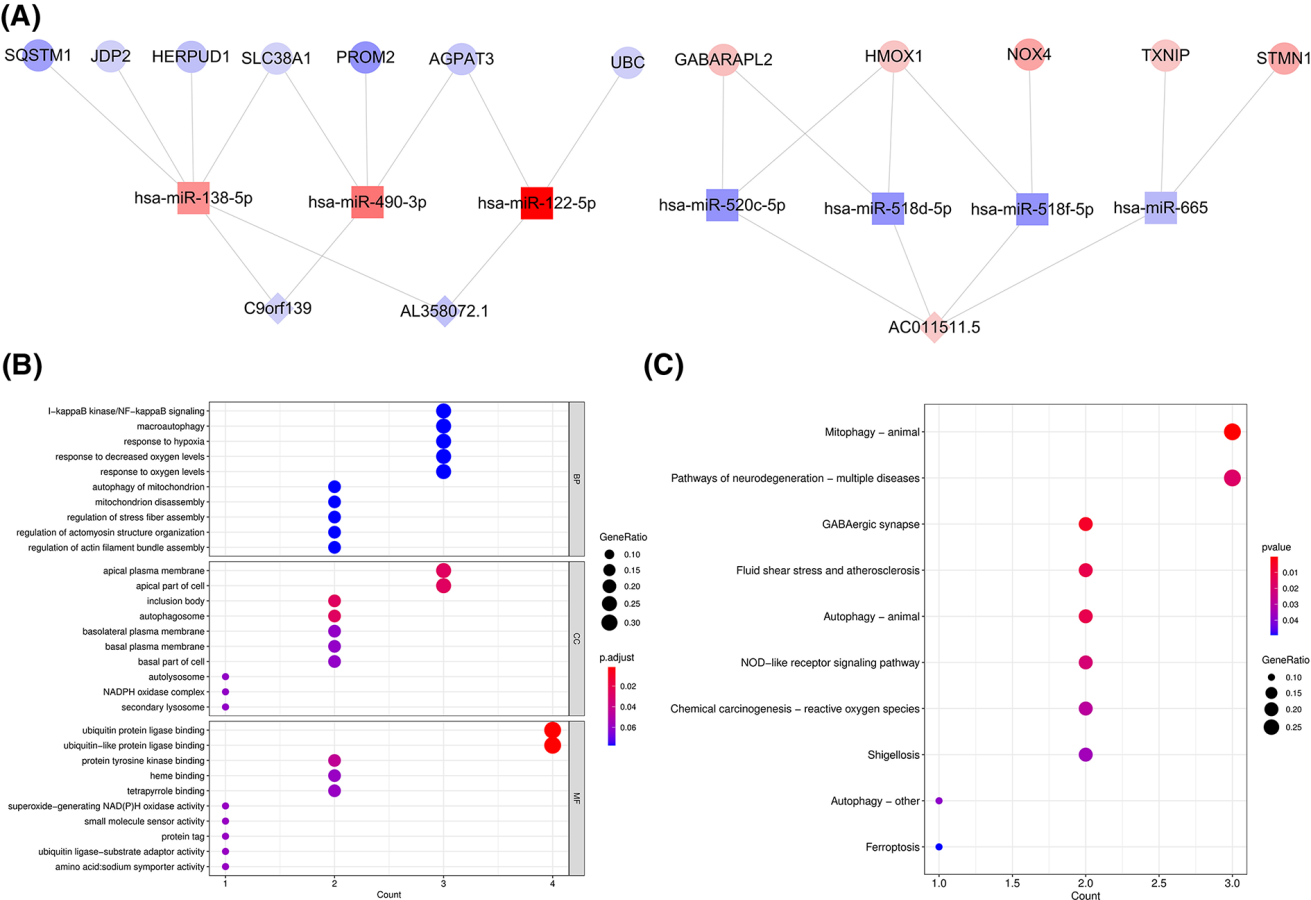
Molecular mechanism of key lncRNA-based subceRNA network (**A**) SubceRNA regulatory network was extracted. The diamond, square and circle indicate lnRNA, miRNA and mRNA, seperately. Blue represents down-regulated genes and red represents up-regulated genes, darker color indicates greater degree the changes have happened of gene expression. (**B**) GO analyses results of DE-FRGs. (**C**) KEGG Pathway analyses results of DE-FRGs. (top10 according to adjusted *P*-value).

Afterward, we interpreted the subceRNA network based on 3 pivotal DE-lncRNAs (Figure 5A), which embraced 22 nodes (12 DE-FRGs, 7 DE-miRNAs, and 3 pivotal DE-lncRNAs) and 25 edges, constituting 21 ceRNA regulatory mechanisms. The lncRNA AC011511.5, which was relatively uplifted in OA, could bind competitively to 4 miRNAs (hsa-miR-520c-5p, hsa-miR-518d-5p, hsa-miR-518f-5p, and hsa-miR-665) to regulate the expression of 5 DE-FRGs, including GABARAPL2, HMOX1, NOX4, STMN1, and TXNIP. The lncRNA AL358072.1, which was characterized by relatively reduced expression in OA, could modulate the expression of 6 DE-FRGs (AGPAT3, HERPUD1, JDP2, SLC38A, SQSTM1, and UBC) through competitive binding to hsa-miR-138-5p and hsa-miR-122-5p. Meanwhile, the expression of AGPAT3, HERPUD1, JDP2, SLC38A, and SQSTM1 could also be mediated by the competitive binding of lncRNA C9orf139 to hsa-miR-138-5p and hsa-miR-490-3p, and their competitive binding could also influence the expression of PROM2. The expression of the 12 extracted DE-FRGs in GSE114007 was consistent with the results of subceRNA network, including that among AGPAT3, HERPUD1, JDP2, SLC38A, SQSTM1 and PROM2 expressed lower in OA samples, in contrast, the expressions of GABARAPL2, HMOX1, NOX4, STMN1 and TXNIP were higher in case samples. There was no significant difference of UBC expression observed between the groups (Supplementary Figure S4).

### Probing the hidden regulatory mechanisms of pivotal DE-lncRNAs-based subceRNA networks in OA

To further investigate the probable molecular mechanisms of the subceRNA network, analogously, we executed a functional enrollment analysis for the 12 DE-FRGs addressed in this network. In the GO-BP category, the results revealed that these genes were inextricably linked to autophagy, response to oxygen, immune-inflammatory response, and apoptotic processes. Additionally, these genes might also provide molecular functions such as ubiquitin (ubiquitin-like) protein ligase binding, protein tyrosine kinase binding, and heme binding in cellular components such as inclusion bodies, apical plasma membrane, and autophagosomes. The top 10 entries of the three categories in the GO system were plotted in Figure 5B, and the detailed GO enrichment results had been synthesized in Supplementary Table S10. In the KEGG enrichment analysis, these genes were relevant to the NOD-like receptor signaling pathway in addition to being engaged in autophagy and disease (atherosclerosis, neurodegenerative and disease, shigellosis) pathways. Moreover, the ‘Ferroptosis’ pathway was inevitably enriched (Figure 5C and Supplementary Table S11). Concordantly, an IPA was conducted against these 12 DE-FRGs suggesting that the ‘Ferroptosis Signaling Pathway’ was prominently activated (Supplementary Figure S5). The above results implied that a subceRNA network based on pivotal DE-lncRNAs might mediate OA progression by registering the ferroptosis, immune-inflammatory response, and oxygen homeostasis.

### Expression of pivotal DE-lncRNAs in OA

**Figure 6:**
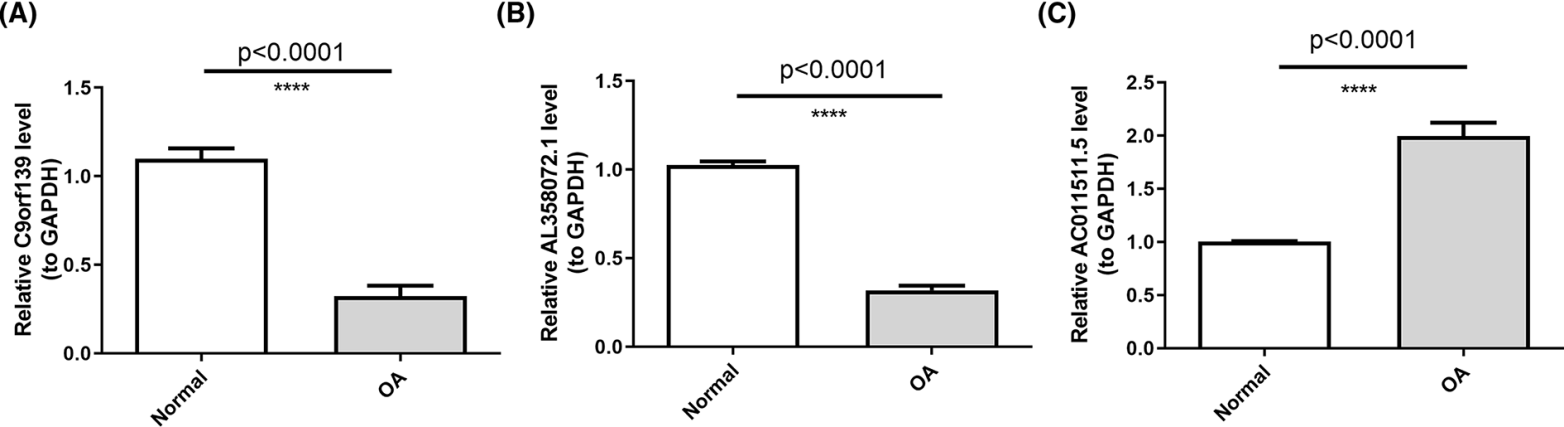
qRT-PCR validation of pivotal DE-lncRNAs between OA and normal controls (**A**) The relative expression of lncRNA C9orf139; (**B**) The relative expression of lncRNA AL358072.1; (**C**) The relative expression of lncRNA AC011511.5.

**Figure 7:**
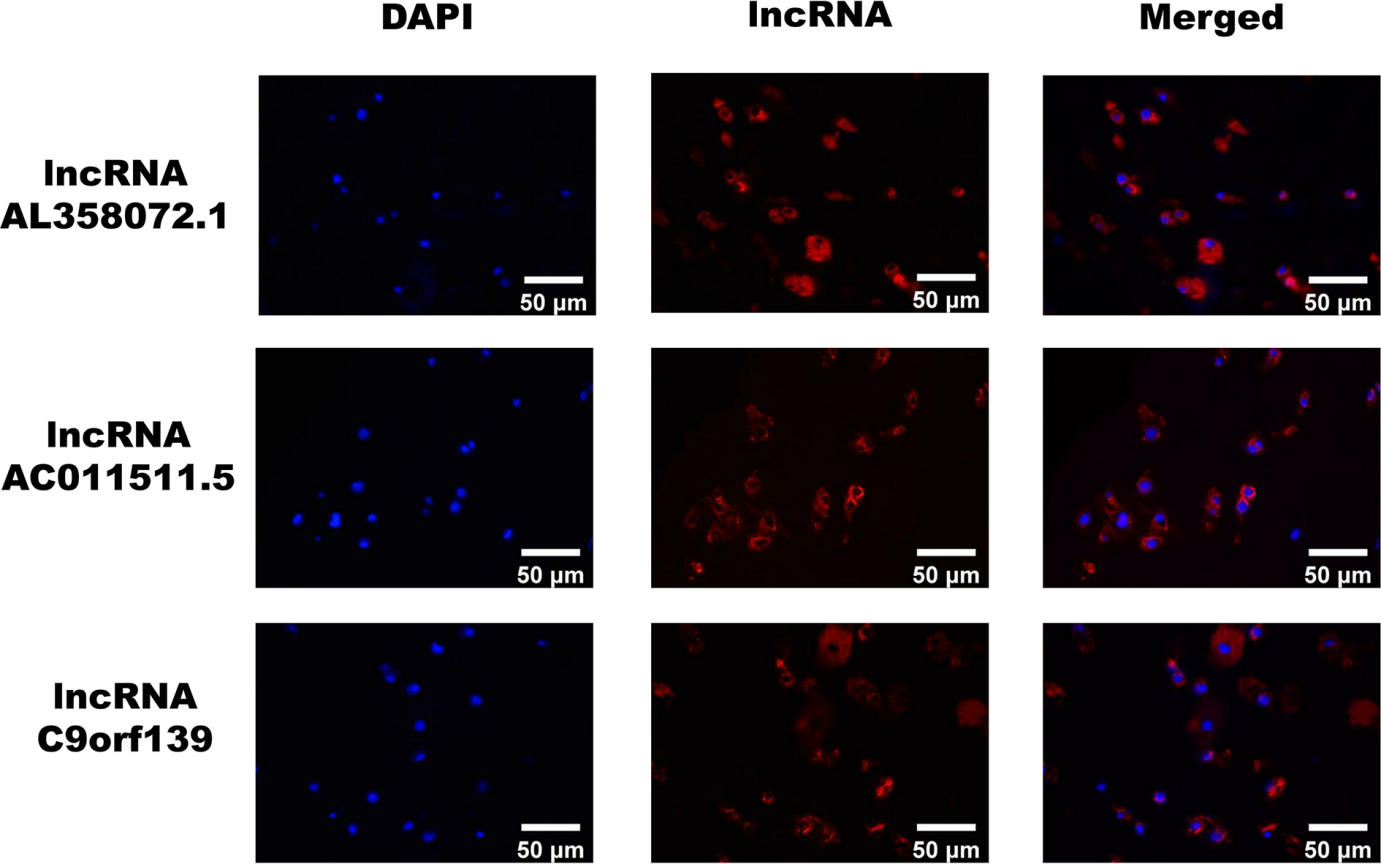
Expression and localization of lncRNA AL358072.1, lncRNA AC011511.5 and lncRNA C9orf139 in OA cartilage tissue

To verify the authenticity of our identified pivotal DE-lncRNAs, we selected 10 cartilage samples from OA patients and 10 cartilage samples from normal subjects for qRT-PCR. The results showed significant differences in the relative expression of all pivotal DE-lncRNAs between OA patients and normal subjects (*P*<0.0001), as shown in Figure 6. compared with the control group, lncRNA C9orf139 and lncRNA AL358072.1 were notably down-regulated in the OA group, whereas lncRNA AC011511.5 was distinctly overexpressed in the OA group, which in accordance with bioinformatics results. To map the location of lncRNAs, we applied FISH technique and the cytoplasmic expression of lncRNA C9orf139, lncRNA AL358072.1, and lncRNA AC011511.5 were detected in OA cartilage tissue, as shown in Figure 7.

## Discussion

Ferroptosis is a cell death process that is morphologically, biochemically and genetically distinct from apoptosis, autophagy, pyroptosis, and various forms of cell necrosis [13]. It has emerged as an effective mechanism for the prevention of several tumors and degenerative diseases, such as Alzheimer’s disease, Parkinson’s disease, and renal degeneration [50]. Recently, there is accumulating evidence that abnormal iron metabolism and ferroptosis are key factors in fostering cartilage degeneration in OA [20,51,52]. Meanwhile, many reports have shown that modification of ferroptosis processes, such as iron overload and oxidative stress, may be an effective strategy for the treatment of OA [53,54]. In recent years, some non-coding RNAs, such as circRNAs, lncRNAs and miRNAs, have also been widely reported to act as ceRNAs to regulate OA occurrence and progression [55]. However, the ceRNA network related to the mediation of ferroptosis by lncRNAs has not been reported in OA. In view of the important roles of ferroptosis and lncRNAs in OA development, the present study constructed a lncRNA-mediated ceRNA network in OA based on ferroptosis-related genes from a bioinformatics perspective, which may provide a new direction for OA diagnosis and treatment.

In our study, we constructed the ceRNA network from differential miRNAs, lncRNAs, and mRNAs based on the data of OA patients in the GEO database, and finally obtained 98 lncRNA-miRNA-mRNA relationships, which contained 26 mRNAs and 20 miRNAs and 20 lncRNAs. To further predict the function of the ceRNA network, KEGG and GO enrichment analyses were used. GO enrichment analysis revealed that a large number of functional entries related to oxidative stress were significantly enriched, such as response to oxidative stress, cellular response to oxidative stress, response to oxygen levels, etc. Oxidative stress is one of the predominant factors in the development of OA [56]. In OA, local inflammatory responses as well as aging or mechanical loading can lead to increased oxidative stress, accumulation of reactive oxygen species (ROS), superoxide anions, hydrogen peroxide (H_2_O_2_), nitric oxide (NO), and peroxynitrite, and concomitant breakdown in the expression of antioxidant enzymes and ROS scavenging systems [56–58]. At the cellular level, oxidative stress leads to mitochondrial DNA (mtDNA) and nuclear DNA damage, lipid peroxidation, alterations in cellular signaling, and epigenetic changes in transcription and gene expression [47–49]. In joints, oxidative stress leads to abnormalities in cartilage and bone metabolism, exacerbating the potential for not only degradation but also overall repair of chondrocytes, osteoblasts and their precursors [47–49]. In chondrocytes, oxidative stress has the potential to interact with multiple cell death mechanisms. By promoting apoptosis, inducing cellular autophagy imbalance, pyroptosis and other mechanisms involved in the pathogenesis of OA [59–61]. Recent evidence suggests that oxidative stress is also engaged in OA chondrocyte ferroptosis [24]. Cell or organelle membranes are particularly prone to ROS damage under oxidative stress due to their high polyunsaturated fatty acid (PUFA) content [62]. Antioxidants such as superoxide dismutase (SOD), catalase (CAT) and glutathione peroxidase (GPX) can minimize or terminate damaging compounds caused by ROS. When adequate redox homeostasis is compromised, cells will undergo lipid peroxidation due to reduced antioxidant activity or a large amount of ROS, eventually leading to oxidative stress-induced ferroptosis [63]. Therefore, our study suggested that the occurrence of ferroptosis in OA might be closely related to oxidative stress, and the regulation of redox homeostasis might improve the ferroptosis of chondrocyte and limit OA progression.

Similar to the GO enrichment results, KEGG enrichment showed that the ‘Ferroptosis’ pathway was inevitably enriched, moreover, some OA-related pathways were significantly enriched, similar to the NOD-like receptor (NLR) signaling pathway. NLRs chiefly induce inflammatory responses and regulate a variety of biological processes, including NF-κB and MAPK-mediated transcriptional pathways, antigen presentation, autophagy, embryonic development and the assembly of cytosolic signaling complexes known as inflammasomes [64]. In specific, studies have shown that NLRP3 plays an inflammatory role in the development of OA [65]. NLRP3 protein expression has been previously reported to be higher in patients with knee OA than in controls [66]. It has been evident that during pyroptosis, NLRP3 can trigger the maturation and secretion of IL-1β and IL-18 as well as tumor necrosis factor α by assembling an intact inflammasome complex, which in turn amplifies the inflammatory response. These factors lead to the release of cartilage-degrading enzymes on chondrocytes, such as aggregated proteoglycanases and metalloproteinases [66,67], which play a key role in the pathogenesis of OA. There is indication that various NLRP3 stimulators enhance intracellular ROS and that enhanced ROS are critical for NLRP3 inflammasome activation [68]. Nuclear factor E2-related factor 2 (Nrf2) plays an important role and is activated in response to ROS to protect cells from inflammation-induced oxidative stress [69]. It was shown that Nrf2 signaling pathway inhibition may be critical for activation of NLRP3 inflammatory vesicles and is involved in the onset and development of OA [66]. Similarly, Yan et al. [67] found that the Nrf2/HO-1 axis inhibits NLRP3 inflammatory vesicle activation, consequently attenuating OA, showing the interaction between the Nrf2 pathway and the NOD pathway in OA. GO functional entries are enriched for a large number of functions centered on intracellular oxidation levels, similarly, KEGG is enriched for the HIF-1 signaling pathway, which is strongly linked to oxygen levels in the organism. HIF is a transcription factor in the cellular environment that is activated in the presence of hypoxia in the organism. The expression of HIF-1α and HIF-2α is altered in osteoarthritic cartilage to mediate the chondrocyte response to hypoxia. HIF-1α is of critical importance in cartilage homeostasis by enhancing ECM synthesis and inhibiting apoptosis, and HIF-1α is used as a survival factor to regulate autophagy and apoptosis [70]. HIF-2α is a potent regulator of autophagy in mature chondrocytes and is catabolic transcription factor in the process of OA [70]. During the development of OA, the activity of HIF-2α and its upstream regulator NF-κB increases [71]. To date, several mechanisms by which HIF-2α regulates OA have been demonstrated. On the one hand, HIF-2α directly induces high expression of catabolic factors, leading to articular cartilage destruction. On the other hand, HIF-2α levels in human and mouse OA chondrocytes are highly correlated with various programmed cell deaths. Meanwhile, HIF-2α enhances Fas-mediated chondrocyte apoptosis and decreases autophagy expression during OA progression [72]. Recently, a novel mechanism of HIF-2α during OA progression was demonstrated to enhance cell death through lipid oxidation, ROS accumulation and ferroptosis regulation [72]. Indeed, there is emerging evidence of an intrinsic link between autophagy and ferroptosis. On the one hand, some studies have identified autophagy as an upstream mechanism to induce ferroptosis by regulating cellular iron homeostasis and cellular ROS production [73]. On the other hand, ferroptosis regulators are also involved in the control of autophagy [74]. These mechanisms may be relevant to the function of the role of the ceRNA network in our study and provide theoretical directions for further experimental exploration.

In our study, lncRNAs were screened based on the topological features of the ceRNA network, and C9orf139, AC011511.5 and AL358072.1 were finally identified as key lncRNAs. Importantly, we verified in human OA cartilage tissue that lncRNA-AC011511.5 was significantly up-regulated and AL358072.1 and C9orf139 significantly down-regulated. The mechanism of action of C9orf139 in OA is unclear. Interestingly, there are a number of studies on the mechanism of action of C9orf139 in other tumors. C9orf139 has been reported to be highly expressed in pancreatic cancer and may serve as a potential diagnostic and prognostic indicator of pancreatic cancer. It promotes pancreatic cancer cell growth by mediating the miR-663a/sox12 axis [75]. Likewise, it was shown that lncRNA C9orf139 regulated the progression of esophageal squamous carcinoma by mediating the miR-661/HDAC11 axis [76]. Moreover, Qin et al. experimentally demonstrated that lncRNA C9ORF139 regulated TAOK1 through sponge miR-24-3P to promote proliferation, invasion and migration of acute myeloid leukemia cells [77]. According to the GPL26963 annotation platform records, AC011511.5 and AL358072.1 are newly identified lncRNAs, and there is no relevant literature report. However, a number of target genes have been reported to be involved in the pathological process of OA. MicroRNA-17-5p was shown to promote osteoarthritis progression through binding to p62/SQSTM1 [78]. Zhang et al. [79] identified GABARAPL2 as a potential target for intervention in OA through biomimetic analysis, and its expression was upregulated in OA tissue samples. It was shown that HMOX1 is highly expressed in synovial membranes of OA patients and OA rat models has counteracted the cartilage damage induced by ROS production, and that inhibition of Nrf2/HMOX1 signaling may promote NLRP3 inflammasome, which in turn promotes OA development [66]. Similar results have been obtained from other studies who suggest that activation of the Nrf2/HMOX1 signaling pathway contributes to the remission of OA [80–82]. Study has shown that NOX4 is overexpressed in OA patients and that NOX4-mediated ROS production has been shown to play a critical role in OA [83]. by constructing a rat model of OA, Liu et al. [84] found that inhibition of NOX4 blocked the effects of USP7 on focal death, ROS production and NLRP3 inflammasome activation. Several studies [85–87] have shown that targeted inhibition of the TXNIP/NLRP3 signaling pathway may also improve the pathological manifestations of osteoarthritis and promote the repair of cartilage damage in OA cell models and mouse models. Taken together, the target genes of these central lncRNAs are closely related to the development and progression of OA. Therefore, our findings may establish a foundation for further discovery of potential diagnostic and therapeutic targets. To further explore the regulatory mechanisms of the three OA-related key DElncRNAS-mediated ceRNA networks, we constructed sub-networks of key lncRNAs and performed functional enrichment analysis of the genes in the sub-networks. Compared with the enrichment results of intact ceRNAs, a large number of oxidative stress-related functional entries as well as oxygen level pathways were again significantly enriched. This part of the enrichment results validates the importance of oxidative stress in the pathological process of OA. In addition, we performed IPA analysis of mRNAs in the ceRNA subnetwork. Classical signaling pathway analysis significantly enriched for 1 pathway and 4 mRNAs associated with the ferroptosis signaling pathway being activated. Consistent with the functional enrichment results, the ferroptosis pathway was significantly activated. Selective activation of the autophagic degradation pathway promotes ferroptosis by increasing iron accumulation causing lipid peroxidation, whereas in this part of the analysis, the autophagic pathway was not significantly enriched, whether this is related to activation of the ferroptosis pathway needs to be further verified. The main core mechanism of organismal cellular defense against ferroptosis is the activation of Nrf2-dependent antioxidant response through transcriptional upregulation of antioxidant or cytoprotective gene expression, and we also observed Nrf2-mediated oxidative stress response in IPA analysis, but due to the small number of genes, it was not shown whether it was activated or inhibited.

However, our study has some limitations, first, the sample size of qRT-PCR is insufficient and further studies are needed to increase the sample size. We were unable to add experimental validation of the relevant target genes due to sampling difficulties. None of the three key lncRNAs we obtained were reported in OA, and the results from publicly available databases and bioinformatics analysis need further biological studies to validate. We will continue to focus on the role of these lncRNAs and further refine cellular and animal experiments to confirm the molecular mechanisms in OA progression.

## Supplementary Material

Supplementary Figures S1-S5

Supplementary Tables S1-S11

## Data Availability

GSE114007, GSE143514, and GSE175960 were downloaded from GEO database (https://www.ncbi.nlm.nih.gov/geo/). The other data used and/or analysed during the present study are available from the corresponding author on reasonable request.

## Abbreviations

CAT: catalase
FISH: fluorescence *in situ* hybridization
GPX: glutathione peroxidase
lncRNA: long non-coding RNA
mtDNA: mitochondrial DNA
NLR: NOD-like receptor
Nrf2: nuclear factor E2-related factor 2
OA: osteoarthritis
PPI: protein–protein interaction
PUFA: polyunsaturated fatty acid
ROS: reactive oxygen species
SOD: superoxide dismutase
