# Supplementary material for: Bioinformatics identification and integrative analysis of ferroptosis-related key lncRNAs in patients with osteoarthritis
Source: Biosci Rep. 2023 Sep 13;43(9):BSR20230255. doi: 10.1042/BSR20230255 (PMC10500229; doi:10.1042/BSR20230255)
Supplement: Supplementary Figures S1-S5 [file BSR-2023-0255_supp.pdf]

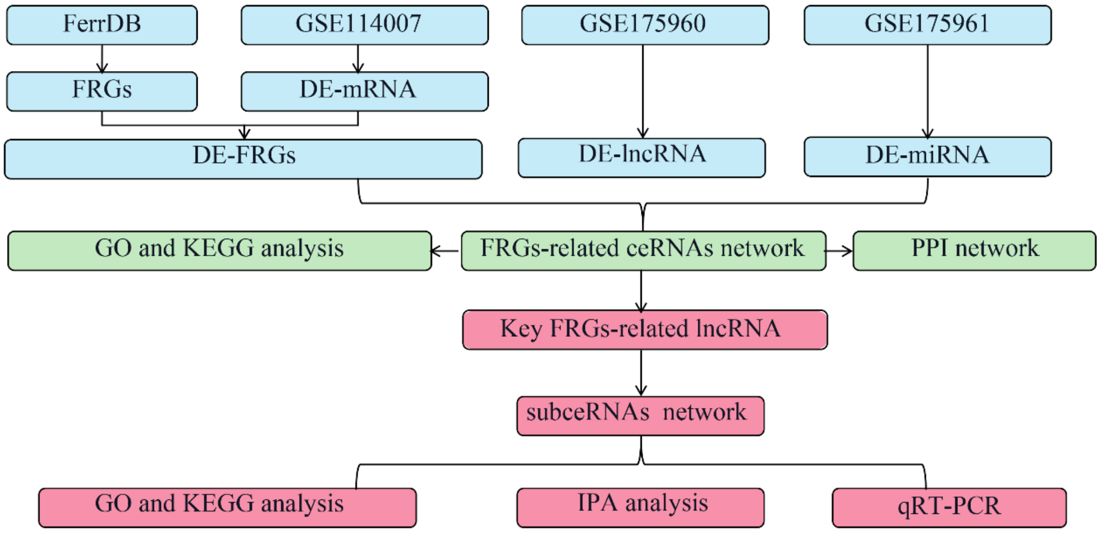

Supplementary Figure 1. The flowchart of this study.

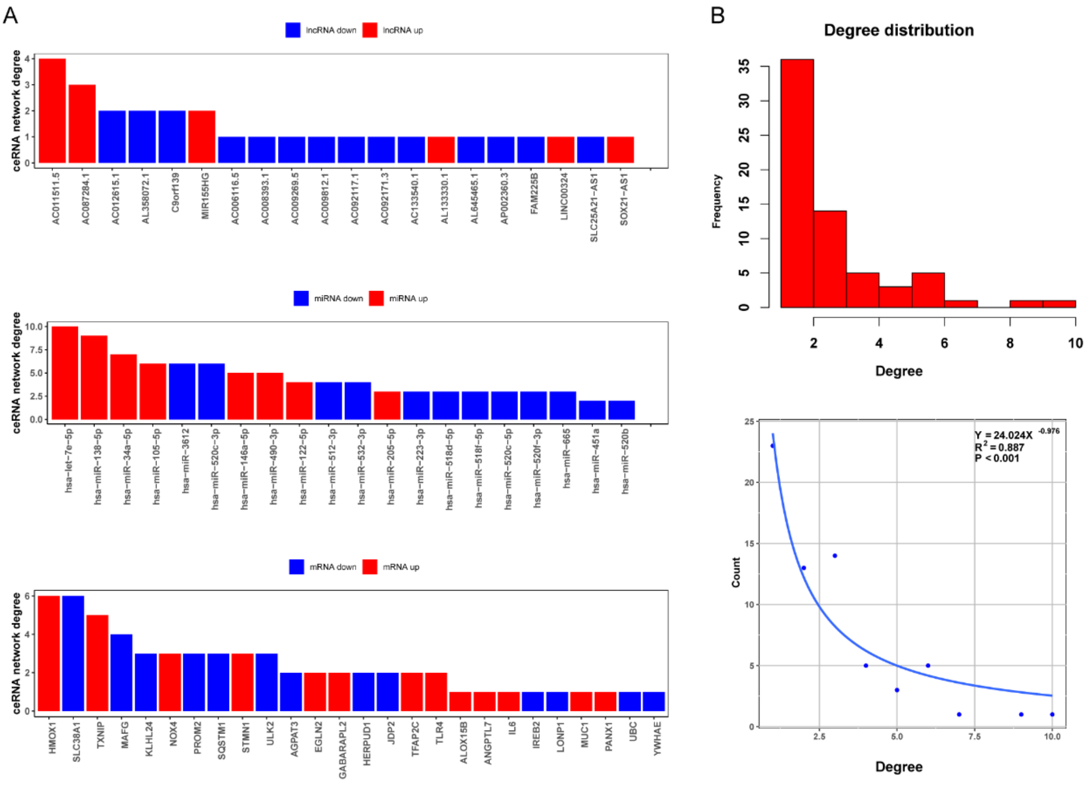

Supplementary Figure 2. The degree characteristics of the nodes in ceRNA network. (A) The degree of the nodes in ceRNA network. (B) The degree distribution of the nodes in ceRNA network.

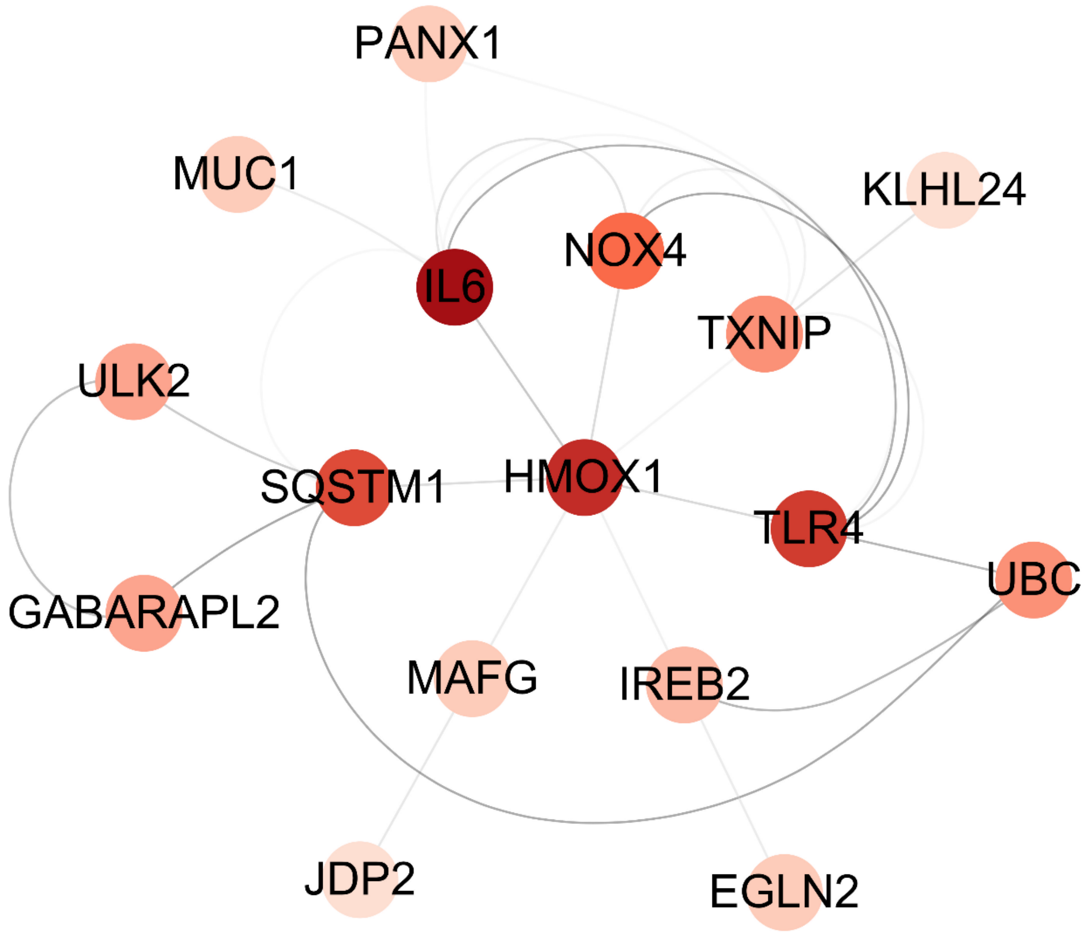

Supplementary Figure 3. PPI network of 26 DE-FRGs extracted from ceRNA network.

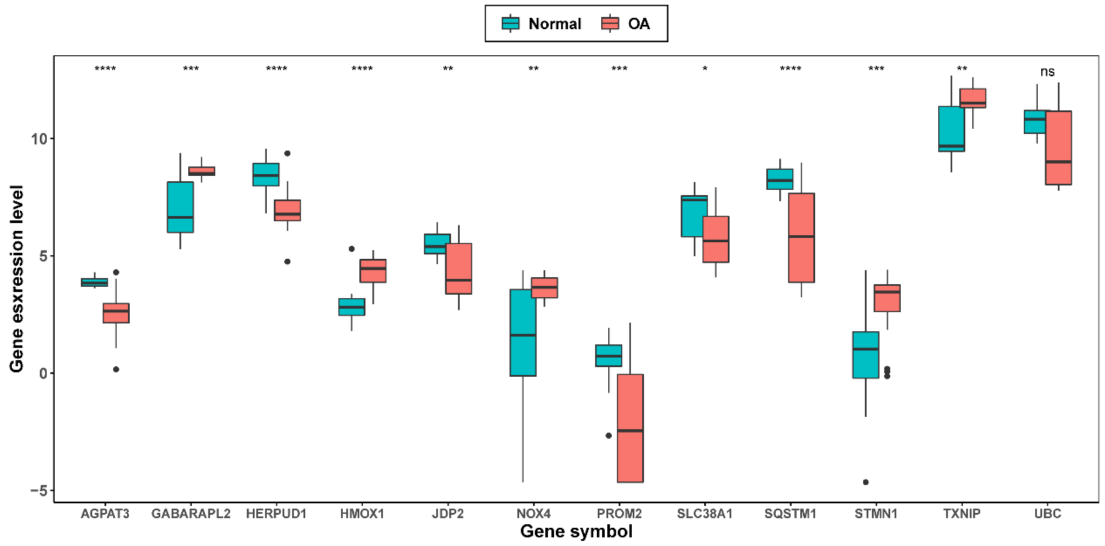

Supplementary Figure 4. The expression levels of 12 DE-FRGs extracted from subceRNA network in GSE114007.

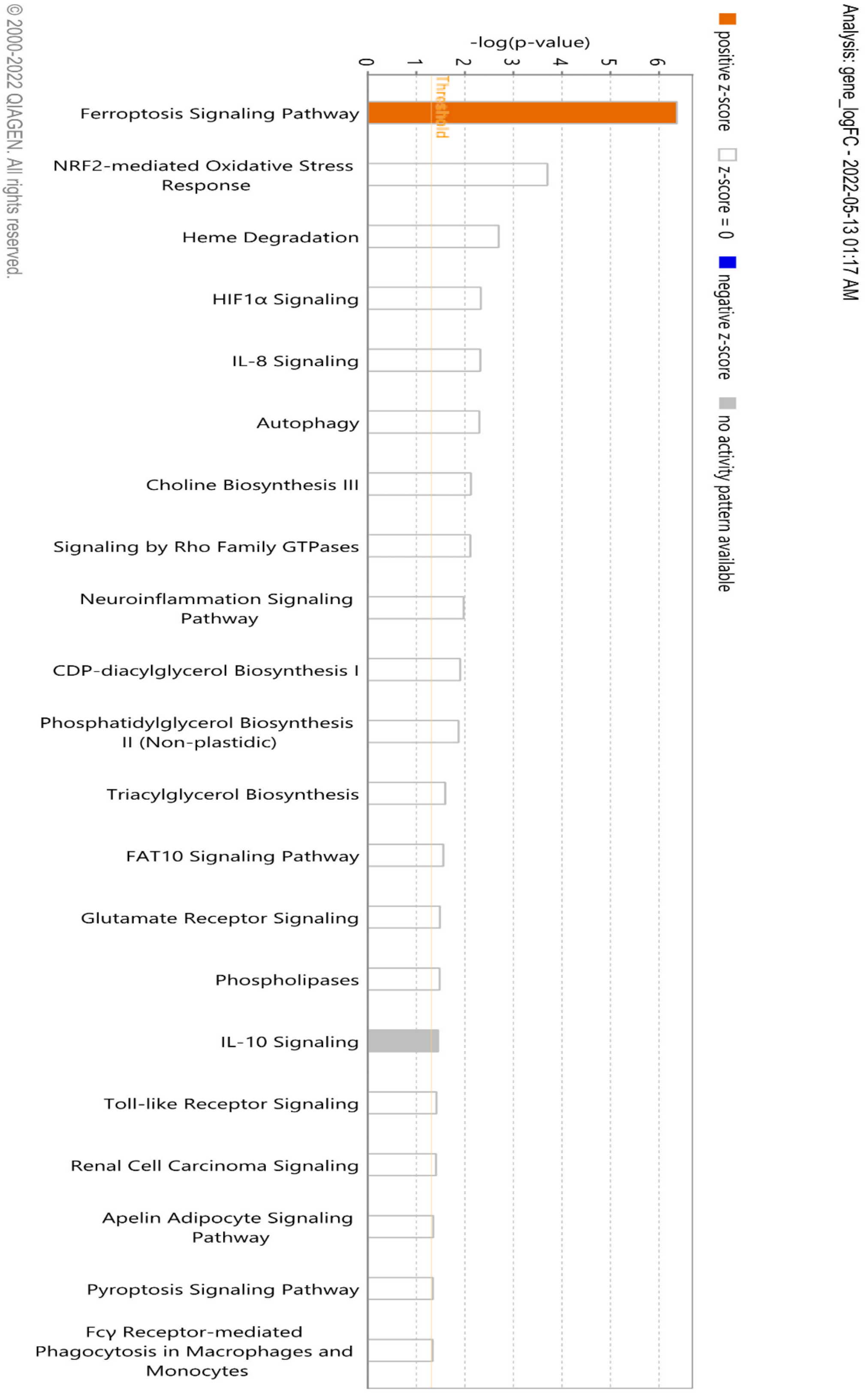

Supplementary Figure 5. IPA analysis of 12 DE-FRGs extracted from subceRNA network.
